# CT features of COVID-19 patients with two consecutive negative RT-PCR tests after treatment

**DOI:** 10.1038/s41598-020-68509-x

**Published:** 2020-07-14

**Authors:** Zhao Fu, Ningning Tang, Yanqing Chen, Longbai Ma, Youyong Wei, Yumin Lu, Kun Ye, Hang Liu, Fen Tang, Guangyi Huang, Yingxia Yang, Fan Xu

**Affiliations:** 1 Department of Radiology, People’s Hospital of Guangxi Zhuang Autonomous Region, Nanning, China; 2 Department of Ophthalmology, People’s Hospital of Guangxi Zhuang Autonomous Region, Nanning, China; 3 Department of Nephrology, People’s Hospital of Guangxi Zhuang Autonomous Region, Nanning, China; 4 Department of Scientific Research, People’s Hospital of Guangxi Zhuang Autonomous Region, Nanning, China; 5 Department of Respiratory, People’s Hospital of Guangxi Zhuang Autonomous Region, Nanning, China

**Keywords:** Diseases, Medical research

## Abstract

The objective of this study is to expound the CT features of COVID-19 patients whose throat swab samples were negative for two consecutive nucleic acid tests after treatment. We retrospectively reviewed 46 COVID-19 patients with two consecutive negative RT-PCR tests after treatment. The cases were divided into moderate group and severe/critical group according to disease severity. Clinical and CT scanning data were collected. CT signs of pulmonary lesions and the score of lung involvement were expounded. Thirty-nine moderate cases and seven severe/critical cases were included. Residual pulmonary lesions were visible in CT images. Moderate patients showed peripheral lesions while severe/critical cases exhibited both central and peripheral lesions with all lobes involvement. Mixed ground glass opacity (GGO) and pulmonary consolidation were noted. A larger proportion of severe patients showed reticular pulmonary interstitium thickening. Air bronchogram, pleural effusion, vascular enlargement, bronchial wall thickening, bronchiectasis, pleural thickening and pleural adhesion were more frequently observed in severe/critical group. The severe/critical group showed higher CT score. Pulmonary lesions persisted even after twice consecutive negative nucleic acid tests. We strongly recommended regular follow-up of CT scans after nucleic acid tests conversion. Evaluation of complete remission should base on chest CT.

## Introduction

Coronavirus Disease-2019 (COVID-19) is an acute infectious disease mainly involving the respiratory system^1^. The highly contagious disease is caused by a novel coronavirus currently termed severe acute respiratory syndrome coronavirus 2 (SARS-CoV-2)^1^. So far to April 9, 2020, 1,479,748 cases of COVID-19 patients and 87,444 deaths are reported. It is a huge strike to human health and draws much attention from countries all over the world.

At present, etiological examinations, including reverse transcription-polymerase chain reaction (RT-PCR) and gene sequencing of sputum, throat swab and lower respiratory tract secretion, are the gold standard for diagnosis of COVID-19^2^. Nucleic acid tests are widely recognized as the primary criteria of discharge after treatment. However, it remains unclear whether damage to the lung have been completely restored when the nucleic acid tests are negative after treatment. Explanation of this issue is essential for determining the timing of treatment termination and isolation release.

Chest computed tomography (CT) provides us a powerful noninvasive mean for the diagnosis and monitoring for COVID-19. Ground glass opacity (GGO) and consolidative opacity involving bilateral and peripheral lung were CT hallmarks of COVID-19 pneumonia^3–8^. It has been reported that CT manifestations vary with the course of disease^8^. However, post-treatment patterns of CT images after nucleic acid tests conversion have not yet been described, which are paramount for not only understanding the pathophysiology but also developing management strategies. In the present study, we assessed chest CT images of COVID-19 patients whose nucleic acid tests were negative after treatment, aimed to provide the most up to date evidence and recommendations for the evaluation of COVID-19 remission.

## Results

### Clinical characteristics

A total of 46 patients were included in this study, including 39 cases with moderate COVID-19 and 7 cases with severe/critical COVID-19 (shown in Table 1). The average age was greater in the severe/critical group than in the moderate group (46.2 ± 13.5 vs 57.9 ± 17.0, P = 0.049). There was no statistical difference in gender between the two groups (P = 0.68).

**Table 1:** Demographic and clinical characteristics of COVID-19 patients with two consecutive negative RT-PCR tests after treatment.

Characteristic	Moderate group n = 39	Severe/critical group n = 7	P value
Age (years)	46.2 ± 13.5	57.9 ± 17.0	0.049
Gender (man)	17 (44%)	2 (29%)	0.68
**Symptoms**			
Fever	4 (10%)	1 (14%)	1.0
Dry cough	5 (13%)	2 (29%)	0.29
expectoration	6 (15%)	1 (14%)	1.0
Chest tightness	2 (5%)	0 (0%)	–
Polypnea	4 (10%)	0 (0%)	–
Fatigue	2 (5%)	0 (0%)	–
Diarrhea	2 (5%)	0 (0%)	–
Throat discomfort	2 (5%)	0 (0%)	–

There was no statistically significant difference in symptoms between the two groups. Only 4 (10%) moderate patients and 1 (14%) severe patient presented with fever on admission. Other clinical symptoms of COVID-19 included dry cough, cough with or without sputum, chest tightness, polypnea, fatigue, diarrhea and throat discomfort.

### CT findings

**Figure 1:**
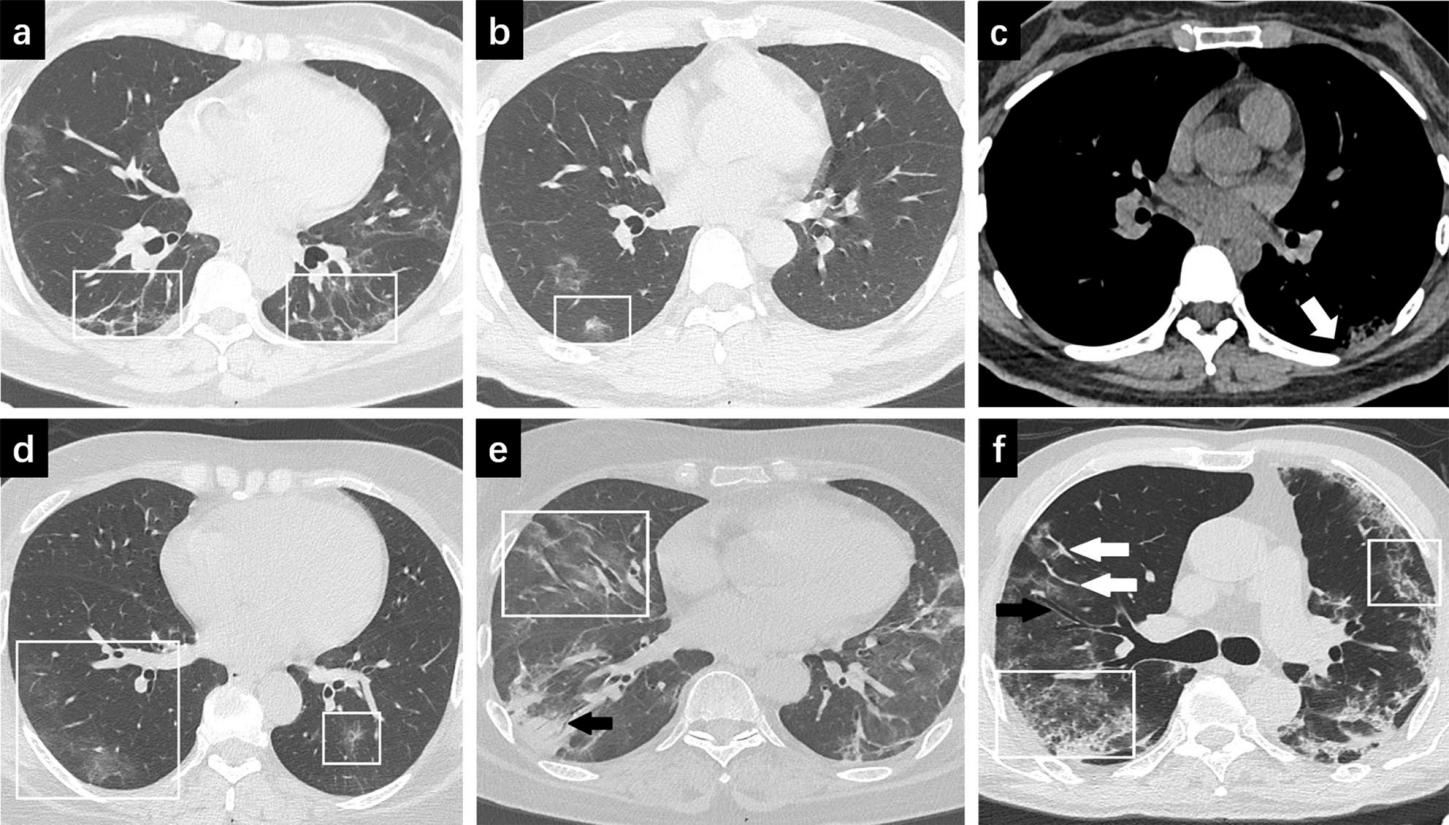
CT images of patients with COVID-19. (**a**) 56-year-old woman with moderate COVID-19. CT image shows pulmonary fibrosis in both lungs (box). (**b**) 37-year-old man with moderate COVID-19. CT image shows mixed ground glass opacity (box). (**c**) 32-year-old woman with moderate COVID-19. CT image shows pleural thickening with pleural adhesion (arrow). (**d**) 50-year-old woman with severe COVID-19. CT image shows ground glass opacities in both lungs (box). (**e**) 59-year-old woman with severe COVID-19. CT image shows ground glass opacities (box) and consolidation with air bronchogram (arrow) in the right lung. (**f**) 65-year-old man with severe COVID-19. CT image shows bronchial wall thickening and bronchiectasis (black arrow). Vascular enlargement is also shown (white arrows). The two boxes show pulmonary interstitium reticular thickening in both lungs.

Residual pulmonary lesions were visible despite two consecutive negative RT-PCR tests (shown in Table 2 and Fig. 1). Multiple lesions were showed in both moderate and severe/critical group (92% vs 100%, P = 1.0). There was a statistically significant difference in lesion distribution between the two groups (P = 0.005). Peripheral lesions were predominant in moderate group (85%) while lesions in both peripheral and central regions were common in severe/critical group (71%). Extensive lesions with five lobes involvement were more significant in severe/critical group than in the moderate group (100% vs 44%, P = 0.01). Mixed ground glass opacity and pulmonary consolidation were more frequently observed in severe/critical group than moderate group (100% vs 41%, P = 0.009; 57% vs 10%, P = 0.012, respectively). Comparison of lesion shape revealed no statistical difference except fan-shaped lesions, which is more common in severe/critical group than moderate group (100% vs 49%, P = 0.014).

**Table 2:** CT features of COVID-19 patients with two consecutive negative RT-PCR tests after treatment.

Feature	Moderate group n = 39	Severe/critical group n = 7	P value
**Number**			
Unique	3 (8%)	0 (%)	–
Multiple	36 (92%)	7 (100%)	1.0
**Distribution**			
Peripheral	33 (85%)	2 (29%)	0.005
Peripheral involving central	6 (15%)	5 (71%)	0.005
**Lobes involved**			
Single lobe	9 (23%)	0 (0%)	–
2–4 lobes	13 (33%)	0 (0%)	–
5 lobes	17 (44%)	7 (100%)	0.01
**Density**			
Ground glass opacity	38 (97%)	7 (100%)	1.0
Mixed ground glass opacity	16 (41%)	7 (100%)	0.009
Consolidation	4 (10%)	4 (57%)	0.012
**Shape**			
Circular	18 (46%)	3 (43%)	1.0
Fan-shaped	19 (49%)	7 (100%)	0.014
Irregular	34 (87%)	7 (100%)	1.0
Pulmonary fibrosis	22 (56%)	5 (71%)	0.682
**Pulmonary interstitium thickening**	28 (72%)	7 (100%)	0.171
Linear	5 (13%)	1 (14%)	1.0
Reticular	23 (59%)	6 (86%)	0.043
**Other findings**			
Air bronchogram	1 (3%)	4 (57%)	0.003
Vascular enlargement	30 (77%)	7 (100%)	0.316
Bronchial wall thickening	3 (8%)	2 (29%)	0.160
Bronchiectasis	5 (13%)	3 (43%)	0.089
Pleural thickening	19 (49%)	6 (86%)	0.106
Pleural adhesion	12 (31%)	5 (71%)	0.083
Pleural effusion	1 (3%)	3 (43%)	0.009
**Total CT score**			
0–5	29 (74%)	0 (0%)	–
6–10	8 (21%)	2 (29%)	0.636
11–15	0 (%)	1 (14%)	–
16–20	2 (5%)	4 (57%)	0.003

A larger proportion of patients showed reticular pulmonary interstitium thickening in severe/critical group than moderate group (86% vs 59%, P = 0.043). Air bronchogram and pleural effusion were significant more frequent within severe/critical group compared to moderate group (57% vs 3%, P = 0.003; 43% vs 3%, P = 0.009, respectively). In addition, vascular enlargement, bronchial wall thickening, bronchiectasis, pleural thickening and pleural adhesion were more frequently observed in severe/critical group, although the differences were not statistically significant (P = 0.316, 0.160, 0.089, 0.106, 0.083, respectively).

Total CT score were significantly higher in severe/critical group compared to moderate group. Most patients in moderate group ranged from 0 to 5 (74%), whereas a majority of patients in severe/critical group ranged between 16 and 20 (57%), which was consistent with the extensive involvement of lesions in severe/critical group.

## Discussion

Achieving two consecutive negative results of nucleic acid tests has currently been recognized as the most important treatment end point for COVID-19 patients. However, our study demonstrated that the pulmonary lesions persisted even after RT-PCR conversion. Multiple lesions such as GGO, pulmonary interstitium thickening and pleural effusion remained common when nucleic acid tests were negative, indicating the presence of dyssynchrony between SARS-CoV-2 nucleic acid tests and chest CT abnormalities.

Multiple lesions with multiple lung lobes involvement were noted in the CT images. The moderate group typically presented with lung peripheral lesions, while the severe group exhibited both peripheral and central lesions. This was similar with other earlier COVID-19 reports^5–7,9–11^. The main pattern of lesions was irregular in this study, which was different from the circular and fan-shaped lesions in early stage of the disease^5,12^. It is probably related to the natural progression of COVID-19. Irregular signs might result from unsynchronized lesion absorption and inter-fusion. In addition, pulmonary fibrosis was observed in five severe patients, which was the result of lesion absorption and recovery.

GGO remained the most common finding after nucleic acid test conversion. In contrast to the early stage of the disease, mixed GGO and consolidation was dominant after treatment^9^. What we have to point out is that air bronchogram could be found in consolidation lesion and some patients had visible bronchial wall thickening and bronchiectasis, which showed inflammation in the bronchi of the lungs.

Pulmonary interstitium thickening is another important sign of COVID-19 pneumonia, which showed more apparent in CT images after nucleic acid tests conversion. Linear pulmonary interstitium thickening was dominated in the early stage, while reticular thickening was dominated after treatment. Pleural thickening and pleural adhesion in COVID-19 patients were rarely reported to date^6,10^. However, visible pleural thickening was observed in half of the moderate patients, of whom a majority presented with pleural adhesion simultaneously. Pleural thickening and pleural adhesion were even more common in severe cases. In addition, small amount of bilateral pleural effusion was observed in one moderate patient and three severe patients. The pleural abnormalities indicated pleural inflammation in COVID-19 patients, especially in severe cases.

The lung scoring method was used to reflect the approximate range of COVID-19 pneumonia. The score of moderate patients were mostly (29 of 39 patients) between 0 and 5, while the score of severe patients were mostly (four of five patients) between 16 and 20. The result showed that the range of residual pulmonary lesion was wider in severe/critical patients than in moderate patients despite two consecutive negative RT-PCR tests.

In clinical practice, two consecutive negative nucleic acid tests were regarded as the most important basis for discharge, however, it should be interpreted with caution, since, in our study, two consecutive negative RT-PCR tests did not signify complete cure of COVID-19 pneumonia. Even though antiviral treatment resulted in progressively lower levels of SARS-CoV-2 until the virus is no longer detectable, the tissue damage caused by overexuberant inflammatory response^13^ was far from complete restoring, instead, aggravation coexists with recovery, as observed in the CT images.

Although we cannot exclude the possibility that laboratorial error could have contributed to some of the inconsistency between nucleic acid tests and chest CT manifestations, the patterns of CT lesions observed in this study suggest that the bulk of the discrepant results reflected the persistence of pulmonary damage despite negative nucleic acid tests. Based upon these results, we would specifically discourage the use of nucleic acid tests results alone for treatment discontinuation and quarantine release decisions, while regular chest CT scans were strongly recommended even after nucleic acid tests conversion to monitor post-treatment cure.

There are some limitations in this study. Firstly, the time from negative nucleic test to CT scanning was not exactly the same, as CT reexaminations were carried out at different time of intervals according to each patient’s condition, which was longer for moderated cases and shorter for severe cases. Secondly, we had not performed further investigation of the pulmonary lesions, due to the lack of inspection equipment in temporary isolation wards. Bronchoscopy, bronchoalveolar lavage and lung biopsy are required to further confirm the nature of the lesions. Thirdly, although a specialized feedback and information sharing system was established between our hospital and other local hospitals to monitor the status of patients after discharge, no re-positive results of nucleic acid tests have been reported up to now. Therefore, this study failed to compare the CT findings of patients with re-positive RT-PCR tests and those with persistent negative RT-PCR tests. We will continue to pay close attention to it.

In conclusion, residual pulmonary lesions remained significant after nucleic acid tests were negative, and became more sophisticated and diverse in comparison with that in earlier stage. These findings provided important insights for pathological mechanism and therapeutic efficacy evaluation of COVID-19, suggesting that chest CT was better than nucleic acid conversion in assessing the final treatment outcomes of the patients. Our results highlighted the importance of using both chest CT and nucleic acid test rather than nucleic acid test alone for monitoring of COVID-19 patients. Evaluation of complete remission should base on chest CT.

## Materials and methods

### Study population

Forty-six consecutive patients were included in this retrospective study. The inclusion criteria were as follows: (1) COVID-19 patients who were treated in the People’s Hospital of Guangxi Zhuang Autonomous Region from February 16, 2020 to March 8, 2020; (2) the throat swab samples were negative for two consecutive nucleic acid tests (obtained at least 24 h apart) after treatment; (3) chest CT was performed after the two negative nucleic acid tests. Patients without CT findings were excluded.

The patients were grouped based on the illness severity defined by the National Health Commission of China^14^. The severe/critical cases met at least one of the following: (1) breathing rate ≥ 30 breaths per min; (2) pulse oximeter oxygen saturation ≤ 93% in a resting state; (3) arteria oxygen tension/inspiratory oxygen fraction ≤ 300 mmHg; (4) respiratory failure (arteria oxygen tension < 60 mmHg when breathing ambient air) occurred and mechanical ventilation required; (5) hemodynamic shock; (6) patients with other organ failure needed intensive care unit monitoring and treatment. Mild patients without CT findings throughout the disease course were not included in the study, so the rest cases were divided in moderate group.

### CT scanning protocol

CT examinations were performed on a 64-detector row SOMATOM go. Top (Siemens Healthineers, Germany) with the following parameters: tube voltage: 120 kVp, tube current with the automatic milliampere technology: 32–200 mAs, pitch: 1.5, tube rotation time: 0.5 s, matrix: 512 × 512, slice thickness: 0.6 mm, reconstruction thickness: 1.0 mm. Unenhanced CT scans were obtained for all patients. Patients were scanned in the supine position, during breath hold. Three chest radiologists with 7 years of experience in thoracic radiology retrospectively reviewed the images independently. Disagreements were resolved through discussion and joint assessment until consensus was reached. The score of lungs was calculated based on the range of lesion involvement: 1–25% involvement is scored as 1 point, 26–50% as 2 points, 51–75% as 3 points and 76–100% as 4 points. Each lung lobe was assessed and total scores were calculated.

### Statistical analysis

Statistical analysis was performed using the SPSS 17.0 software package (SPSS Inc, Chicago, IL, United States). Categorical variables were presented as frequencies or percentages. Continuous variables with normal distribution were presented as the means ± standard deviation (SD) or median (interquartile range, IQR). The Fisher exact test was used for categorical variables. Independent sample *t* test was used for continuous variables with normal distribution. *P*-values < 0.05 were considered statistically significant.

### Ethical approval

The ethics committee of The People’s Hospital of Guangxi Zhuang Autonomous Region approved this retrospective study and waived the requirement for informed consent. This study was conducted in compliance with the Declaration of Helsinki.
